# Strength and Body Composition Changes in Recreationally Strength-Trained Individuals: Comparison of One versus Three Sets Resistance-Training Programmes

**DOI:** 10.1155/2013/615901

**Published:** 2013-09-08

**Authors:** J. S. Baker, B. Davies, S. M. Cooper, D. P. Wong, D. S. Buchan, L. Kilgore

**Affiliations:** ^1^Institute for Clinical Exercise & Health Science, University of the West of Scotland, Hamilton ML3 0JB, UK; ^2^Health and Exercise Science Research Unit, School of Applied Sciences, University of South Wales, Pontypridd CF37 1DL, UK; ^3^Cardiff School of Sport, Cardiff Metropolitan University, Cardiff CF23 6XD, UK; ^4^Human Performance Laboratory, Technological and Higher Education Institute of Hong Kong, Hong Kong

## Abstract

*Purpose.* The purpose of this study was to determine the effects of increasing the volume of weight-training from one to three sets upon body composition and muscular strength. *Methods.* Sixteen male weight-trainers volunteered to act as subjects and were randomly assigned to one of two training groups. Supervised weight-training targeting the upper body was conducted three times per week for eight weeks using one set (*n* = 8) or three sets (*n* = 8) of six repetitions to fatigue. Subjects were measured before and after the training intervention for (1) strength performance (*N* and kg) and (2) adiposity (sum of seven skinfold thicknesses in mm). *Results.* Both training groups improved significantly (20.7%) in terms of muscular strength (*P* < 0.05) with no differences being observed between the one set (21.98% increase) and three set group (20.71% increase) after the training interventions (*P* > 0.05). Significant decreases were also observed for skinfold measures in the one set group (*P* < 0.05). *Conclusions.* One set of high intensity resistance training was as effective as three sets for increasing the strength of muscle groups in the upper body. The one set protocol also produced significantly greater decreases in adiposity.

## 1. Introduction

Strength-training has become one of the most popular forms of exercise for developing musculoskeletal and health-related fitness [1, 2]. From the ages of 13 to 65+ years it remains in the top ten for intended activities. In the USA alone approximately 64,000,000 individuals aged six and above train with barbells and dumbbells and 39,548,000 train with machine weights [3]. The physiological adaptations resulting from a well-designed resistance-training programme can include depending on programme structure increased strength, muscle hypertrophy, increased fat-free mass, increased connective tissue thickness, decreased body fat (with appropriate intervention on diet), and improved physical function [4, 5]. These adaptations occur as a result of alterations in hormone levels, neuromuscular junction activity, motor unit recruitment, and changes in the contractile proteins in muscle [6–8]. However, in designing strength-training programmes there are contrasting and conflicting recommendations regarding the number of weight-lifting sets required to elicit an increase in muscular strength [9, 10]. The most common recommendation for introductory training programmes in healthy populations is to perform multiple sets (at least three) in order to achieve maximal strength gains [11–13]. The main advocates of multiple set training programmes propose that multiple sets are superior for achieving optimal physiological adaptation and that single sets are most appropriate for untrained subjects [1]. Counter to this, Carpinelli and Otto, [9] highlighted that in a survey they had conducted, 33 out of 35 published articles demonstrated no significant difference in increased strength between individuals performing single set training and those performing multiple sets strength-training programmes. This is contradicted by the meta-analysis by Krieger [10] which showed that multiple sets were 40% more effective than single sets. The current guidelines of the American College of Sports Medicine [14] suggest that a single set of exercise is not adequate and 2–4 sets of 8–15 repetitions are preferred.

Another aspect of exercise prescription and strength gain needing consideration is the utility of higher repetitions and lower repetition sets. As indicated by the ACSM position stand of prescribing 8–15 repetitions for strength improvement [14] and experimental findings demonstrating that low repetitions sets are more effective in increasing strength [15], this remains another contentious area of strength training prescription.

This variability of recommendations is largely due to basing exercise prescriptions on research that has been performed on previously untrained subjects [2, 9]. This situation might well create problems in the way in which research outcomes are interpreted because neuromuscular adaptations and learning effects in this population might well influence the accuracy and reliability of the results, and therefore the conclusions drawn from the data may not be reflective of specific adaptations to imposed stresses [16]. The purpose of this study therefore was to investigate the effects of different volumes of strength-training (one set versus three sets) on recreationally strength-trained individuals who had trained with weights for at least one year. In using a sample of subjects drawn from this somewhat trained population, it is proposed that the increases in strength would be more attributable to specific adaptations in physiology rather than the result of learning effects or of neuromuscular adaptations. The hypothesis proposed here is that in this sample population, multiple sets of weighted exercise would elicit larger increases in strength than single set.

## 2. Methods

### 2.1. Sample

Sixteen, healthy male subjects aged between 18 and 21 years who were all recreational strength-trainers (>1 year of experience with the exercises tested) were randomly assigned to either a one-set training group (*n* = 8) or a three-set training group (*n* = 8). Before data collection commenced, the University of Glamorgan Research Ethics Committee approved all elements of the proposed study, and all participants gave written informed consent and volunteered to act as subjects. All subjects were screened to verify that they were not suffering from an injury or disease condition that might affect the study or the subject's ability to participate. All subjects were familiarised with both the training methods and the measurement protocols before data collection, and all subjects maintained their normal diet and refrained from ingestion of additional vitamin and nutritional supplements throughout the training and testing periods. Mean ± *sd* values for age, stature, and mass of the one set training group were 19.8 ± 1.1 years, 1.80 ± 0.08 m, 76.0 ± 9.4 kg; and for the three sets training group they were: 20.0 ± 0.5 years, 1.84 ± 0.06 m, 84.1 ± 11.9 kg. Pretest values for experimental exercises for the 1-set and 3-set groups (bench press 67.24/68.45 kg, biceps curl 41.07/42.97 kg, and shoulder press 42.07/42/68 kg) indicate that the subjects were all able to perform exercises with weights above the standard weight associated with untrained subjects: bench press 55 kg and shoulder press 32 kg [17]. This validated the self-report of subjects' training history of at least one year of strength-training.

### 2.2. Estimation of Body Composition Characteristics

Standard anthropometric measurements were made in accordance with the recommendations for anthropometric assessment [18]. To eliminate interobserver variability only one investigator made the anthropometric measures in the present study. Measurements included stretched stature to the nearest 0.01 m (Seca Wall Mounted Stadiometer, UK) and body mass to the nearest 0.1 kg (Seca Alpha Model 770 Digital Weighing Scales, UK). Body composition characteristics were estimated from the measurement of skinfold thickness, taken on the subjects' right-hand side at the following sites: biceps, triceps, subscapular, supraspinale, abdomen (measured on the subjects left side), pectoral (chest), and mid thigh (anterior). Each skinfold site was measured four times. Skinfold thickness was measured to the nearest 0.1 mm using a set of Harpenden skinfold calipers (Holtain, Crymych, Dyfed, UK). The sum of these seven skinfold thicknesses was used as an estimate of adiposity.

### 2.3. Assessing Strength

In accordance with the ACSM [14] guidelines, the one repetition maximum (1RM) test for muscular strength was used to assess the strength performances of subjects using the following three free-weight barbell exercises: bench press, biceps curl, and shoulder press. Before testing, all subjects were given a demonstration that fully familiarised them with the required procedures. This ensured that all subjects understood the standard techniques and range of motion required for a legitimate repetition. All 1RM tests on all subjects were conducted using Olympic standard free-weights as the resistive loads on the same bar and bench, with all tests being conducted at approximately the same time of day. Subject/equipment positioning (posture and hand grip) and order of exercises were standardized during both the pre and posttests. During the bench press the barbell was required to travel from full elbow extension down to touch the sternum and return to full elbow extension with every repetition. During the biceps curl the barbell was required to travel from full elbow extension to complete elbow flexion and return to full elbow extension with every repetition. During the shoulder press the bar was required to travel from touching the anterior deltoids/clavicle to full elbow extension and return to touching the anterior deltoids/clavicle with every repetition. All exercises were performed at the pace of 2 seconds upward and 2 seconds downward.

### 2.4. The Training Programme

The total duration of the training programme was eight weeks, with the training groups completing three training sessions per week: (1-set group) 6 repetitions × 1 set × 9 exercises × 3 days per week × 8 weeks = 24 sessions and a total of 1,296 repetitions, and (3-set group) 6 repetitions × 3 sets × 9 exercises × 3 days per week × 8 weeks = 24 sessions and a total of 3,888 repetitions. Subjects were required to have completed at least 80% of the training sessions (i.e., 19/24 sessions) in order to be included in the study. Initial training loads were defined as the six repetitions maximum (6RM) or weight that each subject could perform until volitional exhaustion. The initial 6RM load was calculated as 85% of the 1RM load. This procedure allowed for staged and linear increases in resistive loads as the subjects became stronger. Each repetition was required to be performed in a rhythmical fashion through a full range of movement. Two to three minute rest periods were allocated between sets for both training groups in the present study. This is also anecdotally the duration of rest common in the training of competitive weightlifters and powerlifters. Training was performed using free weights as these are considered to elicit a more significant strength response when compared to those from training that uses machines utilizing pulleys, levers, and force converters [19, 20].

During the training period, all training sessions were supervised by at least one of the participating researchers who is certified strength coach as it has been shown conclusively that supervised resistance-training sessions produce better responses from subjects [21]. The strength-training activities performed in the present programme targeted the upper body and included the following nine exercises: bench press, inclined bench press, dumbbell flyes, biceps curl with barbell, biceps curl with dumbbells, hammer curl with dumbbells, seated shoulder press behind neck, lateral raises, and upright row. Therefore, there were 3 exercises for each muscle group. Training sessions for both groups were based on completion of the nine exercises identified. The training programme was kept constant for both the one set (completion of six repetitions on each exercise) and the three sets (completion of 18 total repetitions on each exercise) training groups. Larger muscle groups were always exercised first, with the smaller muscle groups exercised subsequently [5].

### 2.5. Statistical Analyses

The normal distribution and the homogeneity of variance of appropriate data sets were confirmed using the Anderson-Darling test and Levene's test, respectively [22]. It was considered appropriate therefore to test stated hypotheses using parametric statistical methods. A paired sample *t*-test was used to test the null hypothesis (*H*_0_) of no difference between the means for the measured strength performances and sum of skinfolds before (pre) and after (post) the training intervention; *H*_0_: *μ*_pre_ = *μ*_post_ = 0 in both the one set and the three sets training groups. Research hypotheses (*H*_1_) were directionalised, and as a consequence, critical values were established using one-tailed tests (i.e., for strength performances (*N*) *H*_1_: *μ*_post_ > *μ*_pre_; for sum of skinfolds (mm) *H*_1_: *μ*_pre_ > *μ*_post_).

An independent sample *t*-test was used to test the *H*_0_ of no difference between the means for the measured strength performances and sum of skinfolds for the two training groups after the training intervention; *H*_0_: *μ*_1set_ = *μ*_3set_ = 0. Critical values were established using two-tailed tests. Statistical significance was set *a priori* at *P* ≤ 0.05, and in the case of all statistically significant outcomes, the meaningfulness of the differences between the means was expressed with reference to the effect size (*ES, Cohen's d*) and 95% confidence intervals were computed for the relevant mean differences. Finally, the confidence placed in rejecting the *H*_0_ was expressed with respect to the statistical power (%) of the analysis.

## 3. Results

As a result of the training intervention, both groups improved significantly in terms of the means for all the measures of muscular strength (Tables 1 and 2; *P* < 0.05). The 1-set group improved in the bench press by 11.91 kg (17.7%), biceps curl by 8.40 kg (20.5%), and shoulder press by 11.69 kg (27.8%). The 3-set group improved in the bench press by 12.10 kg (17.7%), the biceps curl by 8.00 kg (18.6%), and shoulder press by 9.39 kg (22.0%). Analysis indicates there were no statistically significant differences between the means for the two training groups for any of the measures of muscular strength before or after the training intervention (Table 2; *P* > 0.05). 

After posttraining analyses, no statistically significant differences were observed between the means for the two training groups in terms of either stature or body mass (*P* > 0.05). Interestingly, with respect to sum of skinfolds, there was a significant decrease between the pretest and the posttest for the one-set training group (Table 1; *P* < 0.05) and a significant decrease in sum of skinfolds for the one-set training group (Table 2; *P* < 0.05).

## 4. Discussion

As a result of the eight-week training programme both groups experienced similar and significant improvements in terms of upper-body muscle strength performance (*P* < 0.05). This suggests that eight weeks of linear progressive training can increase upper-body muscular strength by approximately 20% in recreationally strength-trained individuals even when learning effects and early stage neural effects are considered. There were, however, no significant differences observed between groups for muscular strength (*P* > 0.05). This suggests that, given there are 3 exercises for each muscle group, the value of increased set numbers for recreationally strength-trained individuals may not become manifest until after more than a year of training.

Carpinelli and Otto [9] suggested that the crux of the debate concerning the use of one or three sets of resistance-training is between the scientific community and strength trainers who utilise the training methods in the field and that scientific data clearly indicates one set is sufficient. However, Byrd and colleagues [11] are of the opinion that one-set of resistance training is not sufficient to elicit an optimal response in strength gains despite some academic support that favours single set training. With few studies actually using experienced highly trained athletes as subjects, the physiological effects of single and multiple sets on highly conditioned athletes warrant consideration [2, 23]. The most compelling evidence supporting the use of multiple sets in training is the work of Krieger [10] whose meta-analysis on the topic is in opposition to the review by Carpinelli and Otto [9].

As the majority of one and three sets resistance-training studies have considered the effects of training by observing previously untrained subjects, the literature presents data without controlling or at least describing a confounding variable in the experimental design of such studies, training state [24, 25]. Simple description and categorization of training status via subject self-report of months/years of training history cannot adequately enable the researcher to assign subjects into stratifications relative to adaptive physiological status that can affect training outcomes. In this paper we have described subjects as recreationally strength-trained individuals as although they reported training regularly for more than a year—a characteristic used by some researchers to classify subjects as experienced or well trained—the subjects were only capable of lifting weights of a magnitude representative of approximately one year or less. Subjects here would be categorized as high performing new trainees or lower performing novices at entry and high performance novices at exit [17].

Variables such as neuromuscular adaptations and learning effects can be influenced by the number of repetitions performed [26]. As indicated by the present data, it is likely that investigating strength adaptation in well-trained and experienced strength-trainers (intermediate, advanced, or elite trainers per Kilgore, Kilgore et al. [17]) may be required to completely control for the effects of learning and facilitate a more meaningful assessment of the strength gain differences between one-set and three-set training programmes. 

The findings of the present study, and those reviewed by Carpinelli and Otto [9], are suggestive that one set of strength resistance training is just as effective as three sets of training in beginners or recreationally strength-trained individuals. The implications of these findings have a wide range of effects on the prescription of resistance exercise. Within the commercial fitness context, prescribing a single set of exercise across several exercises reduces the time in the gym for a trainee and thus increases the available time for personal trainers or other instructional staff to attend to more paying clients. As observed in this study and in others such as that by Starkey and colleagues [25], the time taken to complete a one-set training programme was significantly lower than that taken to complete three (*P* < 0.05). This approximated to a 66% decrease in exercise time. This reduction in time investment might have positive effects on training programme compliance, reduce injury rates and fatigue, and improve profitability. The overall efficiency of using the single-set method can benefit individuals who desire the health and fitness benefits of resistance training, but might not have the time to devote to multiple-set training programmes, and do not have particularly defined performance and athletic goals. In clinical settings, the consequences of these findings are that they create an opportunity to offer patients lower volumes of training that represents a lower systemic stress than multiple-set training. This may further minimize posttraining pain and risk of provocation of symptoms while delivering the same increases in muscle strength. This has ramifications for elimination of unneeded stress and risk of further injury on potentially sensitive hearts, lungs, bones, joints, ligaments, tendons, and muscles. 

Advantages of one-set training in clinical and commercial fitness environments are apparent. There are also certain circumstances where there are practical implications for the athletes. If a sport or strength and conditioning coach is charged with training beginning or recreationally strength-trained individuals, the positive advantages for using one-set training programmes would include creating strength gain in less time. This would give the coach more available time to schedule developmental training relative to other important aspects of sport performance and conditioning (e.g., endurance work, strategic preparation, or technical skills training). Indeed, there might well be implications to over-training that relate to lower levels of fatigue, tiredness, and muscle injury when one- and three-set training sessions are compared. If one set delivers the same gain as three sets in this population, three sets of training may move these trainees closer to their threshold for displaying over-training symptoms. However, this concept warrants further investigation. 

An unanticipated finding of this study was that there were differences observed between the training groups for the sum of seven skinfold thicknesses (Σ7SF). The Σ7SF differed significantly between the one- and three-set training groups (*P* < 0.05). The 1-set experimental group reduced the sum of seven skinfold thickness to a greater degree than the 3-set experimental group (−14.6 mm versus −7.3 mm). Although the data here clearly demonstrates that strength-training can be viewed as an effective means of subcutaneous fat reduction, the changes noted here suggest that the one-set training group produced the greatest alterations in skinfold thickness. This is surprising given the additional metabolic cost of performing two additional sets of exercise representing 2,592 additional repetitions across eight weeks. There is little scientific research available that states categorically that higher training frequencies produce greater alterations in body composition characteristics [1]. However, lower volume training might maintain protein and muscle glycogen stores, reduce intramuscular damage, and therefore facilitate the capacity of muscle to enhance lean tissue formation. The results of the present study suggest that greater training volumes do not produce more rapid adaptations in body composition characteristics than does training at a lower volume. The training frequency in the present study and the previous studies was the same, that is, 3 times a week, and it is believed that lower session volume may need higher training frequency (and vice versa) in order to maintain the similar effects. The present findings might indicate that during this type of strength-training, a one-set training session of upper-body muscle might allow for the enhancement of lean muscle tissue formation to a greater extent when one- and three-set training sessions are compared. Future studies might benefit from more rigorously controlled dietary reporting or even from controlling dietary consumption by trainees. 

The central finding and value of the present study resides in the similar amount of strength gain elicited by both training organizations of upper-body exercises 1 set and 3 sets. If beginning and recreationally strength-trained individuals have a year or less of training history can obtain equivalent upper-body strength gains and decrease sum of skinfolds while performing a significantly lower volume of strength-training (1,296 versus 3,888 repetitions), the implications are extremely attractive for entry-level coaches, commercial fitness professionals, clinicians, and their trainees.

## Figures and Tables

**Table 1:** Pre and posttest means ± sd for measured strength performances and body composition characteristics from both training groups. Confidence intervals (95%), effect sizes and power (95%) for statistically significant results are also provided.

Variables	Pretest	Posttest	Mean difference	95% confidence interval	Effect size	Power (%)
One-set group						
Bench press (*N*)	659.4 ± 112.7	776.2 ± 121.5	+116.8*	41.2 to 214.6	1.00	61.0
Biceps curl (*N*)	402.8 ± 54.8	485.1 ± 48.0	+82.3*	39.2 to 125.4	1.60	85.0+
Shoulder press (*N*)	412.6 ± 71.5	527.2 ± 74.5	+114.6*	52.9 to 175.4	1.57	85.0+
∑7SF (mm)	76.4 ± 28.8	61.8 ± 19.5	−14.6*	−5.6 to 34.8	0.61	31.7
Three-set group						
Bench press (*N*)	671.3 ± 131.3	789.9 ± 96.0	+118.6*	23.5 to 213.6	1.04	63.6
Biceps curl (*N*)	421.4 ± 44.1	499.8 ± 77.4	+78.4*	−23.5 to 180.3	0.65	34.5
Shoulder press (*N*)	418.5 ± 49.0	510.6 ± 62.7	+92.1*	45.1 to 139.2	1.65	85.0+
∑7SF (mm)	73.5 ± 15.4	66.2 ± 17.9	−7.3	−6.6 to 21.3	0.44	21.4

*Result is statistically significant (*P* ≤ 0.05) on a one-tailed test.

**Table 2:** Means ± sd for measured strength performances and body composition characteristics from both training groups after the eight-week training intervention. Confidence intervals (95%), effect sizes and power (95%) for statistically significant results are also provided.

Variables	One-set training group (*n* = 8)	Three-set training group (*n* = 8)	Mean difference	95% confidence interval	Effect size	Power (%)
Bench press (*N*)	776.2 ± 121.5	789.9 ± 96.0	13.7			
Biceps curl (*N*)	485.1 ± 48.0	499.8 ± 77.4	14.7			
Shoulder press (*N*)	527.2 ± 74.5	510.6 ± 62.7	16.6			
∑7SF (mm)	61.8 ± 19.5	66.2 ± 17.9	4.4*	−24.5 to 15.7	0.24	7.8

*Result is statistically significant (*P* ≤ 0.05) on a two-tailed test.
